# Immunomodulating effects of herbal cough medication - in vitro screening in a TLR7- and TLR8-mediated inflammatory model

**DOI:** 10.1186/s12906-026-05307-4

**Published:** 2026-03-13

**Authors:** Johanna Voigts, Lisa Niederreiter, Roman Huber, Stefanie Kowarschik

**Affiliations:** Department of Internal Medicine II, Centre for Complementary Medicine, Faculty of Medicine, Medical Center – University of Freiburg, Freiburg, Germany

**Keywords:** Phytotherapy, Respiratory diseases, Viral mimicry, Immunomodulation, Toll-like receptor, Inflammation, Ivy, Thyme, *Pelargonium sidoides*, Cineole

## Abstract

**Background:**

The study was conducted to investigate the immunomodulatory effects of herbal cough medications available on the market that contain either whole plant extracts from ivy, thyme, *Pelargonium* or the active plant-derived compound cineole on human lung epithelial cells in order to evaluate their potential in the treatment of viral inflammatory processes.

**Methods:**

Different concentrations of the commercial plant medications (five medications that contained total ivy, thyme or *Pelargonium* extracts and one plant medication that contains cineole) were analyzed for their cytotoxicity and immunomodulatory potential on the human epithelial lung cancer cell line A549 and the hTERT-immortalized cell line NuLi-1. Viability was tested using WST-1 assays. AnnexinV/PI staining and flow cytometry analysis were used to investigate apoptosis and necrosis. A549 and NuLi-1 cells were stimulated with toll-like receptor (TLR)7- and TLR8-agonists, mimicking viral infection. Cytokine secretion was analyzed using the bead-based LEGENDplex™ assay.

**Results:**

Non-toxic concentrations of the ivy-, thyme- or *Pelargonium *- containing medications, but not the medication containing cineole, significantly reduced the secretion of inflammatory cytokines and chemokines after the stimulation of TLR7 and TLR8 in A549 and NuLi-1 cells.

**Conclusion:**

Medications derived from ivy, thyme and *Pelargonium* extracts exhibit anti-inflammatory properties in a model mimicking viral infection in human lung epithelial cells.

## Background

Viral respiratory tract infections like the flu, induced by influenza A, are associated with high morbidity and mortality rates every year. There are almost one billion known cases of influenza infections every year worldwide, of which 3 to 5 million have severe symptoms [1]. Common symptoms of influenza infections include cough, fever, sore throat, headache and a runny nose. Symptoms usually persist for one to two weeks [2]. However, in severe cases, hyperinflammatory processes in the lung and dysregulated immune responses occur and can result in death [3]. During ongoing influenza infection, chemokines and cytokines are produced to fight the invading virus. The first line of defense is the respiratory tissue [4]. Cells of the respiratory epithelium play a central role in the cytokine and chemokine response and recruitment of immune cells. Among the key molecular players are toll-like receptors (TLRs), a family of pattern recognition receptors that detect conserved viral or bacterial structures. They are an important part of the innate immune system and also expressed by respiratory epithelial cells. The endosomal TLR7 and TLR8 sense single-stranded ribonucleic acid (ssRNA) of e.g. severe acute respiratory syndrome coronavirus 2 (SARS-CoV-2) or also influenza A. The activation of TLR7- and TLR8-mediated downstream pro-inflammatory signal pathways results in the expression of pro-inflammatory cytokines and chemokines such as interleukin (IL)-6, IL-8 or tumor necrosis factor (TNF)-α [5].

The host inflammatory response to viral infections is a driver of symptoms and disease progression and was intensively studied during the SARS-CoV-2 outbreak [6, 7]. Experimental models have demonstrated that a reduction of cytokine release and consecutively, a decreased infiltration of innate immune cells reduce mortality in mice. Celecoxib and mesalazine are known to downregulate the expression of pro-inflammatory cytokines, that promote inflammation. In a study done with BALB/c mice, a combination of the synthetic drugs zanamivir, celecoxib and mesalazine increased the survival following influenza H5N1 infection [8]. The use of synthetic drugs to treat respiratory infections helps to control and modulate the inflammatory response against the invading virus. Besides synthetic drugs, the use of herbal remedies for the treatment of respiratory diseases has become more popular. Herbal remedies can be used as complementary therapies, supporting treatment outcomes.

Herbal remedies have been used to treat respiratory infections for centuries in all parts of the world. They are a rich source of bioactive substances and many of them interact on inflammatory pathways [9]. There are numerous herbal preparations available on the market that contain either whole plant extracts or one or more active plant-derived compounds. The mode of action of herbal medications in viral lower respiratory tract infections is multifaceted. They have been shown to have direct anti-viral, anti-inflammatory, spasmolytic and secretolytic effects [10–13]. Herbal medications made from ivy, thyme, *Pelargonium sidoides* and cineole alleviated symptoms and reduced the duration of lower respiratory tract infections in placebo controlled clinical trials [14–18]. *P. sidoides* has demonstrated broad immunomodulatory and anti-viral effects in in vitro and in vivo studies, including the modulation of cytokine response and an enhancement of innate immune functions. A clinical trial showed that patients who received 800 mg/ml of *P. sidoides* three times a day showed a change in IL-6 and IL-15 in serum and nasal epithelium [19]. Further clinical studies have investigated ivy preparations with respect to their effects on cough symptoms. Ivy preparations were shown to improve cough symptoms compared to placebo, which was evaluated in a clinical trial. An in vitro study done by Čolić and colleagues showed the impact of an ivy extract on cells of the adaptive immune system and its clear anti-inflammatory effect [20]. A treatment of different human respiratory cell lines showed a reduced release of pro-inflammatory cytokines IL-1 and 89 by inhibiting the nuclear factor kappa-light-chain-enhancer of activated B-cells (NF-κB) pathway. Besides these, also the secretion of the mucosal protein mucin-5AC was reduced [21]. Further evidence from preclinical studies is available for other herbal remedies. A treatment of guinea pigs with cineole showed a reduced level of the inflammatory markers TNF-α and IL-1β. Cineole also prevents the reduction of the mucociliary clearance, which supports the removal of pathogens [22]. Although the therapeutic effects of these herbal medications have been shown in clinical trials, the underlying immunomodulatory mechanism – especially in the context of virus-induced activation of the immune receptors TLR7 and TLR8 – remains largely unexplored. This gap of knowledge is significant, as a balanced immune response is crucial to improving outcomes in viral respiratory infections. While a robust anti-viral response is needed to control infections, excessive or dysregulated inflammation is associated with tissue damage and disease exacerbation.

We investigated whether common herbal cough medications used to treat lower respiratory tract infections can modulate the release of inflammatory cytokines and chemokines. Specifically, we hypothesized that these medications modulate the release of pro-inflammatory cytokines and chemokines following stimulation of TLR7 and TLR8, potentially promoting a balanced immune reaction. While numerous studies have described the clinical efficacy and pharmacological properties of these herbal medications in respiratory tract infections, detailed data on their ability to modulate of TLR7- and TLR8-mediated signaling in respiratory epithelial cells are lacking. The work contributes to refining the scientific rationale behind the use of these widely applied plant-based remedies and helps to identify their capacity to fine-tune host`s inflammatory responses against invading viruses. The aim of this study was to evaluate whether commonly used herbal cough remedies can modulate TLR7- and TLR8-mediated inflammatory responses in respiratory epithelial cells, thereby providing a scientific basis for their use in balancing host immune reactions during viral infections.

## Methods

### Clinical trial number

Not applicable.

### Used herbal cough medications containing extracts or active plant-derived compounds

Commonly available herbal cough medications containing whole herbal extracts or active plant-derived compounds, along with their solvents, concentrations and manufacturers are shown in Table 1. The commercial medications are registered as drugs, according to the German drug law, or as supplements. Each herbal cough medication was diluted 10-, 100- and 1000-fold in the corresponding culture medium before cells were treated in the different assays.

**Table 1:** Used herbal cough medications

Trade name	Compounds	Solvent	Pharmacy central number	Manufacturer
Bronchipret^®^ liquid TE (BRO)	Ivy, thyme	Ethanol	05566226	Bionarica SE, Germany
Prospan^®^ cough drops (PRO)	Ivy	Ethanol	08585951	Engelhard Arzneimittel GmbH & Co.KG, Germany
Soledum^®^ capsule forte (SOL)	Cineole	For assays capsule solved in sodium chloride	00744255	Klosterfrau Healthcare Group, Germany
Tussamag^®^ concent cough syrup (TS)	Thyme	Glycerol/ Ethanol	07632499	Ratiopharm GmbH, Germany
*Thymus vulgaris* mother tincture (TM)	Thyme	Ethanol	04240741	Ceres Heilmittel GmbH, Germany
Umckaloabo^®^ drops (UMC)	*Pelargonium sidoides*	Ethanol	01062032	Dr.Willmar Schwabe GmbH & Co. KG, Germany

### Cell culture

A549 (ATTC number: ATCC^®^ CCL-185™) and NuLi-1 (ATTC number: ATCC^®^ CRL-4011™) were obtained from ATCC (Manassas, Virginia). A549 cells were cultivated in Dulbecco’s Modified Essential Medium (DMEM, w glucose 4.5 mg/ml, w/o sodium pyruvate, Gibco by Thermo Fischer Scientific, Waltham, USA) supplemented with 10% fetal calf serum (Anprotec, Bruckberg, Germany) and 1% penicillin/streptomycin (100 U/ml each, Sigma Aldrich, St. Louis, Massachusetts, USA). NuLi-1 cells were grown in Bronchial Epithelial Cell Growth Medium BulletKit (Lonza, Basel, Switzerland) supplemented with 1% penicillin/streptomycin. For cultivation, culture flasks were coated with collagen (60 µg/ml, Sigma-Aldrich, St. Louis, United States).

### Cell viability

To analyze cell viability, 2 × 10^4^ cells/ml were seeded and allowed to adhere overnight. Cells were then treated with the indicated dilutions of the herbal medications, staurosporine (10 µM; inhibition control, dissolved in DMSO, Sigma Aldrich) or the corresponding solvent controls. After 72 h, cells were washed with phosphate buffered saline (PBS, w/o Mg^2+^ and Ca^2+^; Gibco by Thermo Fischer Scientific) and incubated for 20 min at 37 °C in DMEM (w/o phenol red, Gibco by Thermo Fischer Scientific) containing 5% water-soluble tetrazolium 1 (WST-1) solution (Roche, Indianapolis, USA). The metabolic conversion of the WST reagent was analyzed using a plate reader (Tecan Reader Infinite M 200, Tecan, Männedorf, Switzerland) at 450 nm and 620 nm as reference wavelength.

### Apoptosis/necrosis

Cells were seeded in a concentration of 1 × 10^5^ cells/ml one day prior to treatment. On the following day, they were treated with different dilutions of the herbal medications, staurosporine (10 µM; apoptosis control), Triton X-100 (0.5%; necrosis control, Sigma Aldrich, St. Louis, USA) or the corresponding solvent controls. After 72 h, cells were collected, washed with PBS and centrifuged (300 x g for 5 min, RT) before they were stained with the Annexin V-FITC apoptosis detection kit (eBioscience, Frankfurt, Germany) according to the manufacturer’s protocol. Samples were analyzed using a flow cytometer (FACS LSR Fortessa Instrument; BD Biosciences, Franklin Lakes, NJ).

### Toll- like receptor (TLR) 7 and TLR8 stimulation

The stimulation of TLR7 and TLR8 receptor signaling was performed as described previously [23]. In brief, cells were seeded at a density of 2.5 × 10^5^ cells/ml and cultured overnight. Afterwards, cells were stimulated with interferon-α (IFN-α, 5 × 10^4^ U/µl, Merck KGaA, Darmstadt, Germany) for 6 h, followed by the RNA analogues ssPolyU (5 µg/ml; Invivogen, San Diego, USA) and loxoribine (1 mM; Invivogen, San Diego, USA). RNA analogues were either already pre-coated in lipid vesicles (in the case of loxoribine) or they were pre-treated for 30 min with Lipofectamine-2000 at 37 °C. Treatment with the herbal medications was applied subsequently. After 24 h, cells were used for further analysis.

### Detection of cytokine release

Cytokine release was analyzed using the LEGENDplex™ Human Anti-Virus Response Panel (BioLegend, Amsterdam, The Netherlands) according to the manufacturer’s manual. Cells were first seeded in a density of 2.5 × 10^6^ cells/ml, cultured overnight and treated to mimic a viral infection as described in section TLR7 and TLR8 stimulation. Afterward, cells were treated with the highest non-toxic concentration of the herbal medications, solvent controls, parthenolide (20 µM; anti-inflammatory control; Sigma Aldrich, St. Louis, Massachusetts, USA) or phorbol-12-myristate-13-acetate (PMA; 100 nM; pro-inflammatory control; Sigma Aldrich, St. Louis, Massachusetts, USA). Analysis of secreted cytokines was done using flow cytometry (FACS Fortessa, Becton Dickinson, Franklin Lakes, USA). The resulting data were analyzed with the corresponding LEGENDplex™ Data Analysis Software Suite (BioLegend in collaboration with Qognit, San Carlos, USA).

### Data analysis

One-way or two-way ANOVA was used to determine statistical significance, followed by Dunnett’s post hoc pairwise comparisons. Values are presented as mean ± standard deviation (SD) for the indicated number of independent experiments. The asterisks represent significant differences from controls (*p < 0.05; **p < 0.01; ***p < 0.001; ****p < 0.0001). For statistical analyses, GraphPad Prism 9.0.0 software (GraphPad, San Diego, USA) was used.

## Results

### Effect of the different herbal medications on cell viability

**Fig. 1:**
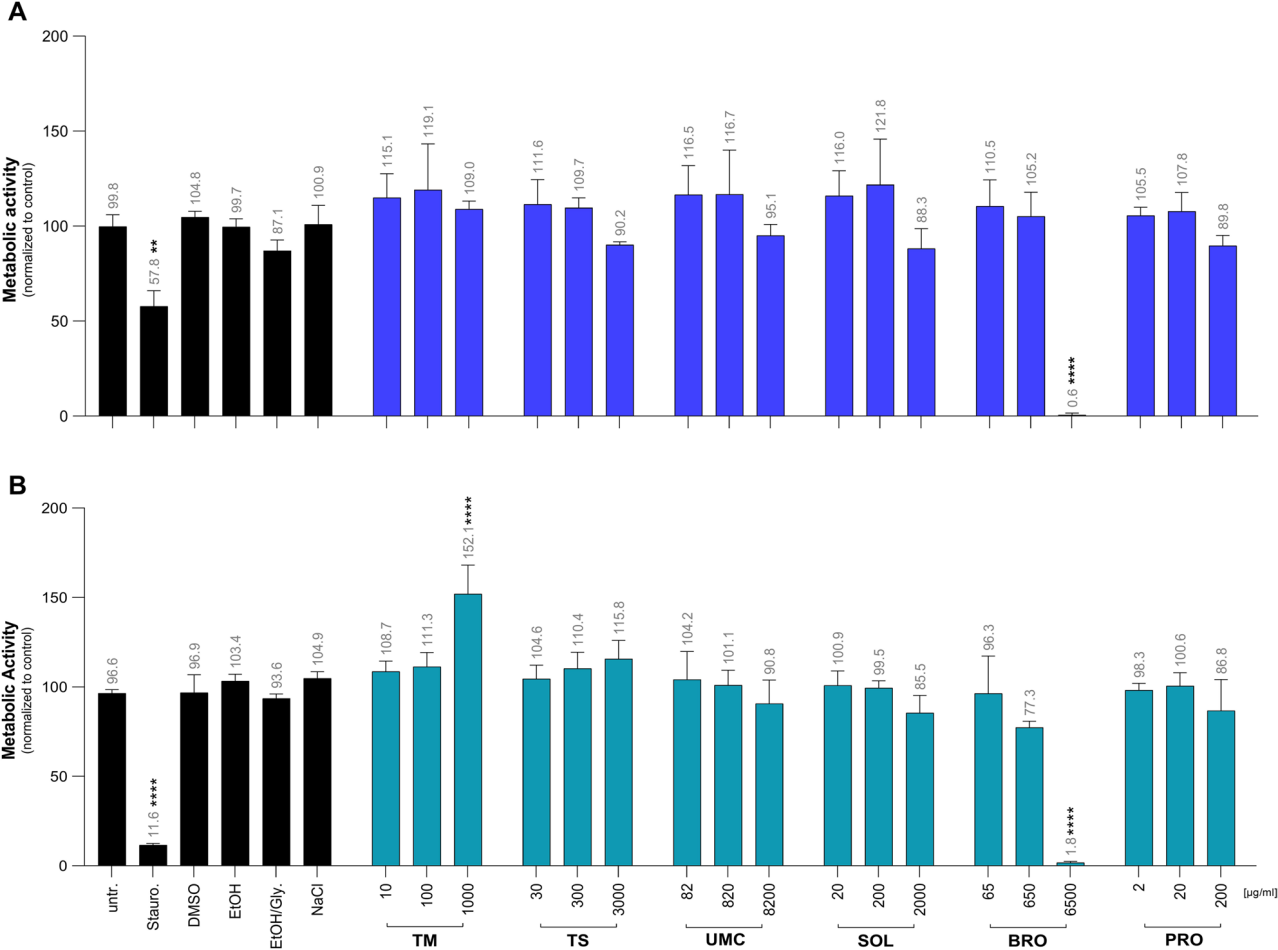
Effects of herbal preparations on metabolic activity. A549 (**A**) and NuLi-1 (**B**) cells were incubated for 72 h with increased concentrations of *Thymus vulgaris* mother tincture (TM), Tussamag^®^ concent cough syrup (TS), Umckaloabo^®^ drops (UMC), Soledum^®^ capsule forte (SOL), Bronchipret^®^ liquid TE (BRO) and Prospan^®^ cough drops (PRO). To determine the metabolic effects, cells were stained using water-soluble tetrazolium reagent, analyzed at a wavelength of 450 nm and compared to the untreated (untr.) control. Staurosporine, (Stauro., 10 µM) served as a positive control, whereas dimethylsulfoxide (DMSO, 1%), ethanol (EtOH, 0.7%), EtOH/glycerol (EtOH/Gly., 0.1%/0.85%) and sodium chloride (NaCl, 0.09%) served as solvent controls. Numbers indicate the mean percentage of viable cells. Data represent the mean ± SD of three independent experiments. **p < 0.01; ****p < 0.0001

Seven different herbal medications were tested for their potential to affect metabolic activity in cells using a WST-1 assay. Staurosporine reduced the viability of A549 (57.8% remaining viability) and Nuli-1 cells (11.6% remaining viability), whereas the solvent controls ethanol, DMSO, NaCl and the ethanol/glycerol mixture did not affect the metabolic activity (Fig. 1). Except for the highest concentration of the BRO (6500 µg/ml; 0.6% remaining viability for A549 and 1.8% remaining viability for Nuli-1 cells) extract, the metabolic activity was not influenced by the medications (Fig. 1).

### Determination of apoptotic or necrotic effects of the herbal medications

**Fig. 2:**
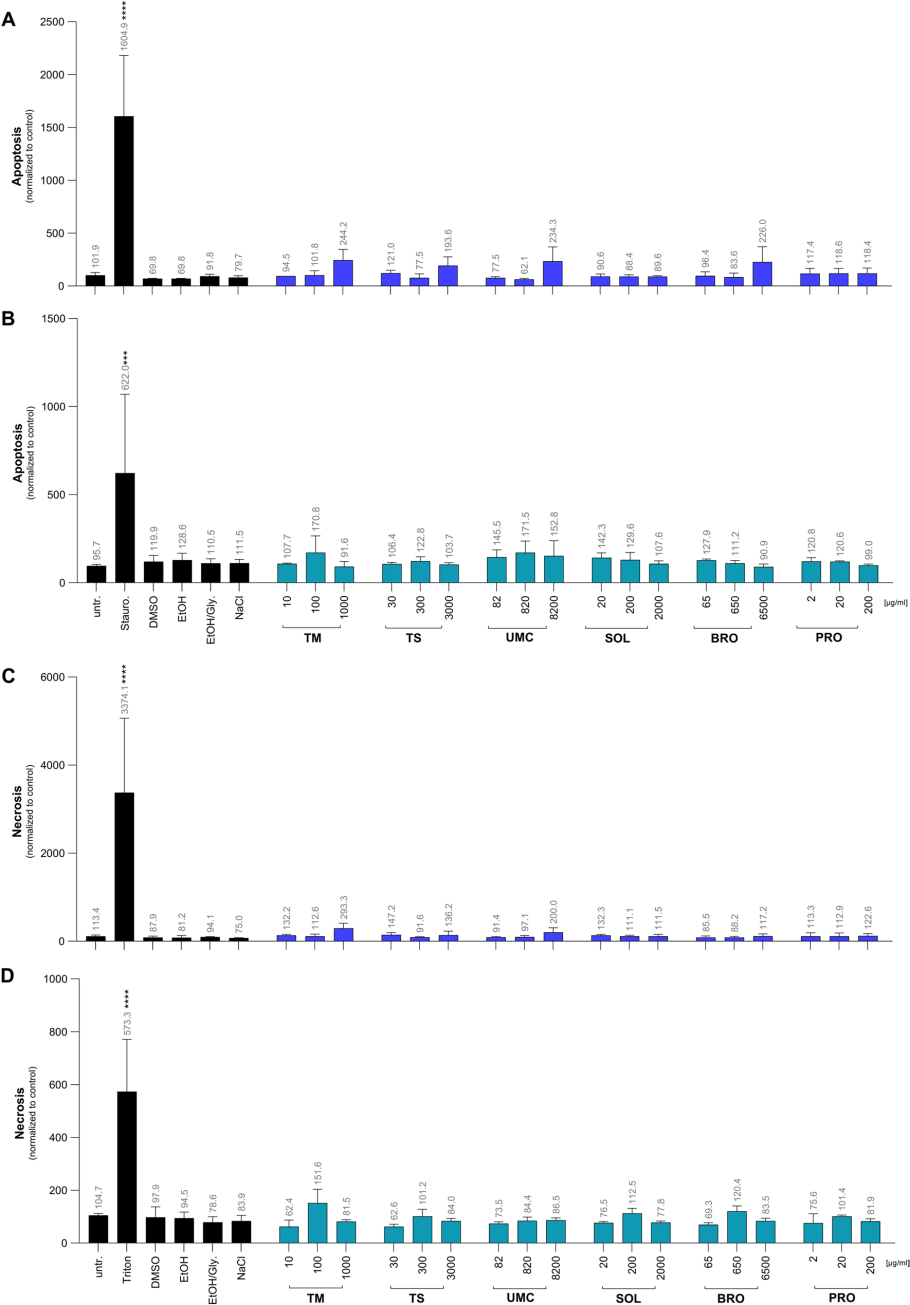
Induction of apoptosis in A549 (**A**) and NuLi-1 cells (**B**) as well as necrosis in A549 (**C**) and Nuli-1 cells (**D**). Cells were incubated for 72 h with increased concentrations of the indicated extracts: *Thymus vulgaris* mother tincture (TM), Tussamag^®^ concent cough syrup (TS), Umckaloabo drops (UMC), Soledum capsule forte (SOL), Bronchipret ^®^ liquid TE (BRO) and Prospan cough drops (PRO). To measure apoptotic and necrotic cells, they were stained and analyzed via flow cytometry and compared to the untreated (untr.) cells. Staurosporine, (Stauro., 10 µM) served as a positive control for apoptosis and Triton X-100 (Triton, 0.5%) served as a control for necrosis. Dimethylsulfoxide (DMSO, 1%), ethanol (EtOH, 0.7%), EtOH/glycerol (EtOH/Gly., 0.1%/0.85%) and sodium chloride (NaCl, 0.09%) served as solvent controls. Numbers indicate the mean percentage of apoptotic or necrotic cells. Data represents the mean ± SD of three independent experiments. ****p < 0.0001

To assess whether the herbal medications induce apoptosis or necrosis, we used annexin V/PI staining. Untreated cells, as well as the cells treated with the solvent controls ethanol, DMSO, NaCl and the ethanol/glycerol mixture, showed no effect on apoptosis or necrosis (Fig. 2). Staurosporine induced apoptosis in A549 (1604.9%) and NuLi-1 (622.0%) cells, whereas Triton X-100 induced necrosis in both cell types (3374.1% for A549 and 573.3% for Nuli-1 cells; Fig. 2). None of the tested herbal medications induced significant levels of necrosis or apoptosis (Fig. 2).

### Inflammatory response of A549 and NuLi-1 cells after TLR7 and TLR8 stimulation

**Fig. 3:**
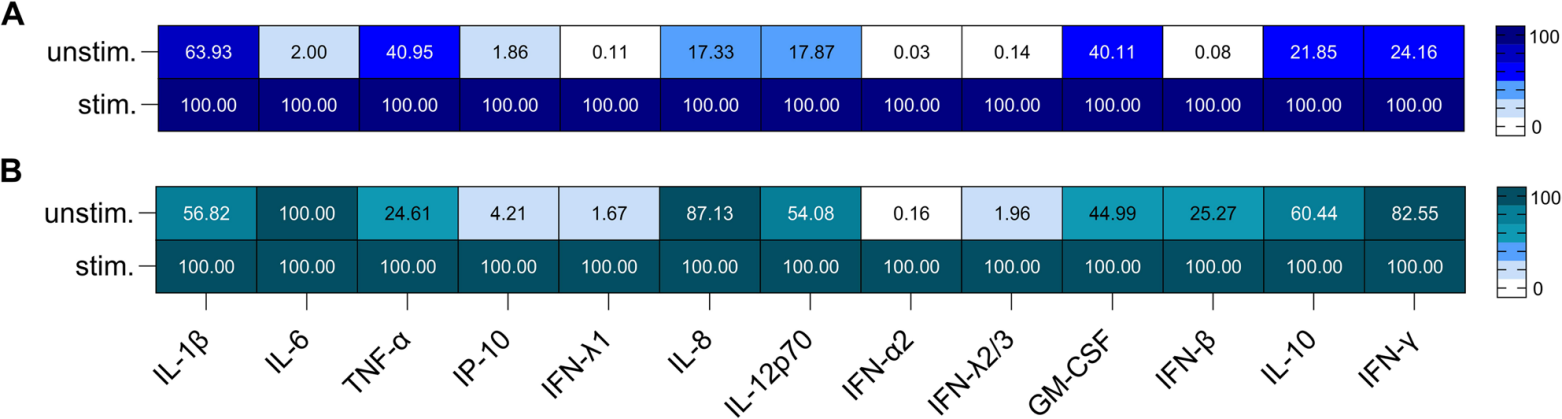
Inflammatory response after virus-like stimulation for A549 (**A**) and Nuli-1 cells (**B**). Cells were left unstimulated (unstim.) or stimulated (stim.) with interferon (IFN)-α (5 x 10^4^ U/µl) and polyuridylic acid (5 µg/ml) and loxoribine (Loxo., 1 mM). Interferon (IFN)-γ, interleukin (IL)-10, IL-6, IL-12p70, IFN-α2, granulocyte-macrophage colony-stimulating factor (GM-CSF), IFN-λ2/3, IL-8, IL-1β, tumor necrosis factor (TNF)-α and interferon-gamma induced protein 10 kD (IP-10) were analyzed from supernatants using flow cytometry. Data represents the mean of three independent experiments.

Because A549 and NuLi-1 cells differ in their origin, we investigated whether they also exhibit distinct cytokine and chemokine responses following TLR7 and TLR8 stimulation. Therefore, we stimulated A549 and NuLi-1 cells with IFN-α to boost the inflammatory response and with the RNA analogues loxoribine and polyuridylic acid (PolyU) to activate TLR7 and TLR8. We analyzed 13 different cytokines and chemokines in A549 and NuLi-1 cells, which play a role in viral infections. For A549, there was an increase for all 13 analytes after TLR7 and TLR8 stimulation compared to unstimulated cells: IL-1β (36%), IL-6 (98%), TNF-α (59%), IP-10 (98%), IFN-λ1 (100%), IL-8 (83%), IL-12p70 (82%), IFN-α2 (100%), IFN-λ2/3 (100%), GM-CSF (60%), IFN-β (100%), IL-10 (78%) and IFN-γ (76%) (Fig. 3A). NuLi-1 cells did not show a significant alteration in IL-6, IL-8 and IFN-γ levels between the unstimulated and stimulated conditions, whereas all other parameters displayed significant changes: IL-1β (43%), IL-6 (0%), TNF-α (75%), IP-10 (96%), IFN-λ1 (98%), IL-8 (13%), IL-12p70 (46%), IFN-α2 (100%), IFN-λ2/3 (98%), GM-CSF (55%), IFN-β (75%), IL-10 (40%) and IFN-γ (17%) (Fig. 3B).

### Immunomodulatory capacity of the different herbal medications

**Fig. 4:**
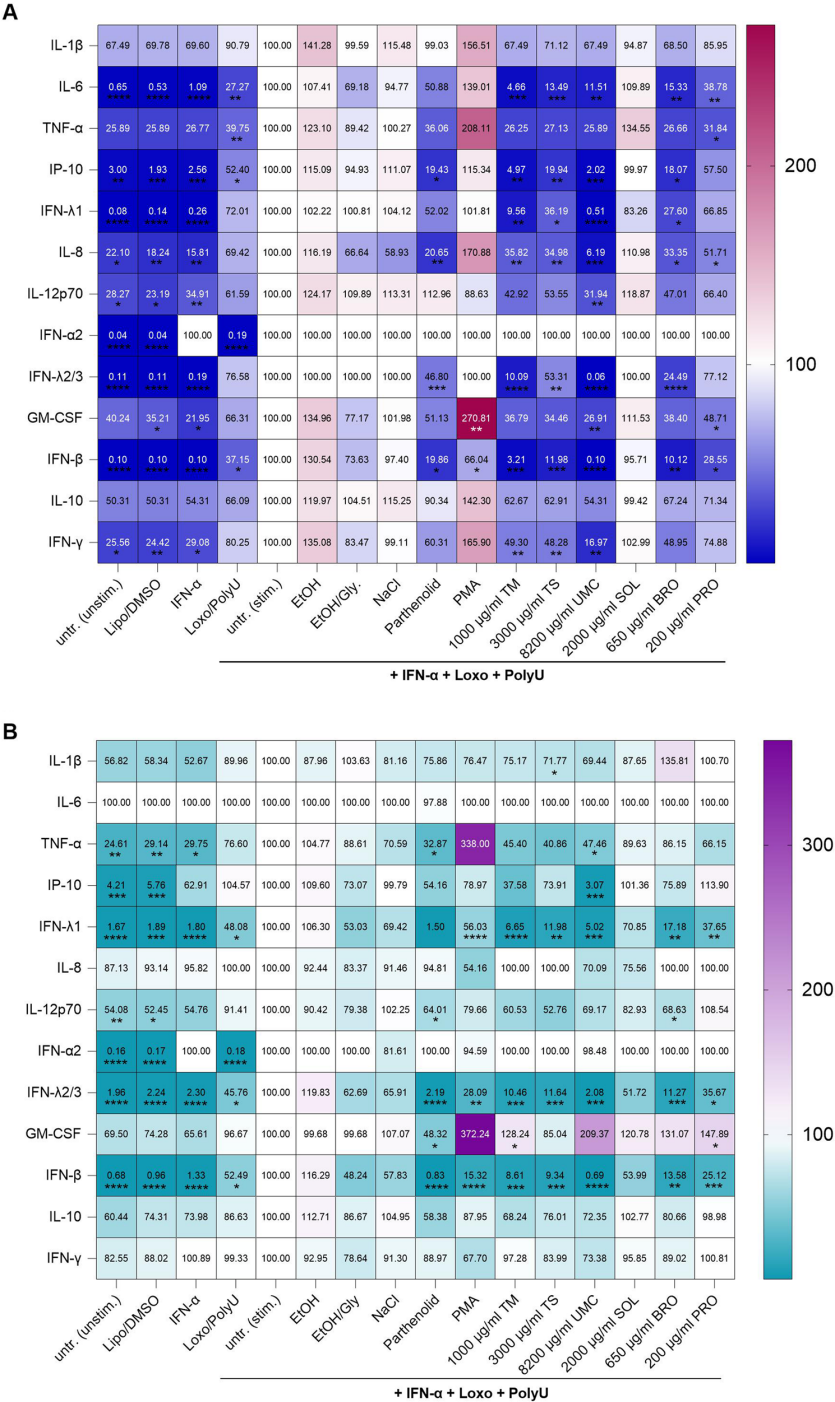
Inflammatory response after virus-like stimulation for A549 (**A**) and Nuli-1 cells (**B**) after incubation with parthenolide (20 µM; anti-inflammatory control), phorbol-12-myristate-13-acetate (PMA; 100 nM; pro-inflammatory control), *Thymus vulgaris* mother tincture (TM, 1000 µg/ml), Tussamag^®^ concent cough syrup (TS, 3000 µg/ml), Umckaloabo^®^ drops (UMC, 8200 µg/ml), Soledum^®^ capsule forte (SOL, 2000 µg/ml), Bronchipret^®^ liquid TE (BRO, 650 µg/ml) and Prospan^®^ cough drops (PRO, 200 µg/ml). Analysis of interferon (IFN)-γ, interleukin (IL)-10, IL-6, IL-12p70, IFN-α2, granulocyte-macrophage colony-stimulating factor (GM-CSF), IFN-λ2/3, IL-8, IL-1β, tumor necrosis factor (TNF)-α and interferon-gamma induced protein 10 kD (IP-10) from supernatants using flow cytometry. Cells were left unstimulated (unstim.), treated with the solvent control (DMSO, 1%), ethanol (EtOH, 0.7%), EtOH/glycerol (EtOH/Gly., 0.1%/0.85%), sodium chloride (NaCl, 0.09%), Lipofectamine (Lipo., 1.88 µl/ 100 µl), IFN-α (5 × 10^4^ U/µl) alone, single-stranded polyuridylic acid (PolyU, 5 µg/ml) and loxoribine (Loxo., 1 mM) or the combination of IFN-α, ssPolyU and loxoribine. Numbers indicate mean percentage of measured cytokines and chemokines. Data represents the mean ± SD of three independent experiments * *p* < 0.05, ** *p* < 0.01, *** *p* < 0.001, **** *p* < 0.0001

After analyzing cytotoxicity, we were interested in the immunomodulatory effect of the different plant extracts in an influenza A infection mimicry system. Therefore, we stimulated A549 and NuLi-1 cells with IFN-α to boost the inflammatory response and with the RNA analogues loxoribine and PolyU acid to activate TLR7 and TLR8. Afterwards, cells were treated with the highest non-toxic concentration of the different herbal medications. In A549 cells, the following cytokines and chemokines were inhibited by at least one of the tested herbal medications compared to the stimulated control: IL-6 (TM 95%, TS 87%, UMC 88%, BRO 15%, PRO 61%), TNF-α (PRO 68%), interferon-gamma induced protein 10 kD (IP-10; TM 95%, TS 80% UMC 98% BRO 82%), IFN-λ1 (TM 90%, TS 63%,UMC 99%, BRP 72%), IL-8 (TM 65%, TS 65%, UMC 94%, BRO 66%, PRO 49%), IL-12p70 (UMC 68%), IFN-λ2/3 (TM 90%, TS 47%, UMC 100%, BRO 75%), granulocyte-macrophage colony-stimulating factor (GM-CSF; UMC 73%, PRO 51%), IFN-β (TM 97%, TS 88%, UMC 100%, BRO 90%, PRO 71%), IFN-γ (TM 51%, TS 52%, UMC 83%) (Fig. 4A). No significant alterations were observed for IL-1β, IFN-α2 or IL-10 (Fig. 4A). In NuLi-1 cells, none of the tested plant medications significantly affected IL-6, IL-8, IFN- α2, IL-10 or IFN-γ (Fig. 4B). IL-1β (TS 28%), TNF-α (UMC 52%), IP-10 (UMC 97%), IFN-λ1 (TM 93%, TS 88%, UMC 92%, BRO 83%, 62%), IL-12p70 (BRO 31%), IFN-λ2/3 (TM 89%, TS 88%, UMC 98%, BRO 88%, PRO 64%), IFN-β (TM 91%, TS 91%, UMC 99%, BRO 86%, PRO 75%) were significantly downregulated compared to the stimulated cells by at least one of the tested substances in NuLi-1 cells. GM-CSF was by upregulated by TM (28%) and PRO (48%) (Fig. 4B). Soledum (SOL) did not show any cytokine or chemokine alteration in A549 and NuLi-1 cells (Fig. 4).

## Discussion

The respiratory epithelium is part of the innate immune system and represents the first line of defense against invading pathogens, such as viruses or bacteria. Cells of the respiratory epithelium produce inflammatory cytokines and chemokines to recruit immune cells to fight pathogens. Inflammation is necessary for fighting invading pathogens, but a strong inflammatory response is also the reason for more intense symptoms and disease burden. Reducing inflammatory processes during ongoing viral infections helps to improve disease burden and outcome [24]. During viral infections, a complex network of cytokines orchestrates the balance between the anti-viral defense and inflammation-induced damages. Pro-inflammatory cytokines such as IL-1β, IL-6 and TNF-α initiate and amplify early immune responses by promoting leukocytes recruitment. Chemokines such as IL-8 and IP-10 attract neutrophils and T cells to the infected tissue, enhancing viral clearance but also contributing to local inflammation. Type I and II interferons limit viral replication, upregulate anti-viral genes, activate natural killer cells and promote macrophage activation. However, uncontrolled or prolonged cytokine release can lead to severe tissue injury, vascular leakage and multi-organ dysfunction [22].

In our in vitro study, we analyzed different herbal medications regarding their capability to reduce inflammatory cytokines and chemokines in a virus mimicry system by stimulating the ssRNA sensing receptors TLR7 and TLR8. Both TLRs are present in the lung and mediate the activation of inflammatory signal pathways. For our experiments, we used A549 cells, a human lung epithelial cell line derived from a lung carcinoma and NuLi-1 cells which are normal human lung bronchial epithelial cells immortalized by dual retroviral infection. The use of these different cell lines provides insights into the inflammatory response. Nuli-1 cells are closer to the in vivo situation compared to A549 cells, because they are not isolated from tumor tissues [25, 26]. Among lung epithelial cell lines, A549 cells are commonly used; however, their cancerous origin makes them less representative of in vivo conditions. NuLi-1 cells were selected for their ability to retain characteristics from normal lung cells [27].

First, we were interested in the cytotoxicity of the different plant medications to determine non-toxic concentrations in the used respiratory cell lines. Regarding cytotoxicity, only very high concentrations of the thyme/ivy medication Bronchipret^®^ (6500 µg/ml) altered the metabolic activity in A549 or Nuli-1 cells without inducing cell death. None of the tested plant medications caused apoptosis or necrosis. Once non-toxic concentrations of the herbal preparations had been determined in the used cell lines, we analyzed the inflammatory response induced by TLR7 and TLR8 activation.

A stimulation of TLR7 and TLR8 in A549 and NuLi-1 cells induced an inflammatory response. In A549 cells, an increase of all 13 cytokines and chemokines (IL-1β, IL-6, TNF-α, IP-10, IFN-λ1, IL-8, IL-12p70, IFN-α2, IFN-λ2/3, GM-CSF, IFN-β, IL-10 and IFN-γ) was observed. The stimulation of TLR7 and TLR8 in Nuli-1 cells only induces a significant upregulation of IL-1β, TNF-α, IP-10, IFN-λ1, IL-12p70, IFN-α2, IFN-λ2/3, GM-CSF, IFN-β and IL-10, but not for IL-6, IL-8, and IFN-γ. The findings for NuLi-1 cells are in line with previous studies, where they also showed that IL-6 and IL-8 production could not be further increased through TLR7 and TLR8 stimulation [26]. The different origin of the cells also explains their different behavior concerning their cytokine response after TLR7 and TLR8 activation.

Regarding immunomodulation, except for the cineole-containing Soledum, all medications inhibited the TLR7-, TLR8-mediated inflammatory signals in A549 and Nuli-1 cells. TNF-α, as well as type I IFNs, IFN-α and IFN-β, have important functions at early stages of infection. They induce the expression of anti-viral proteins and recruit immune cells [28, 29]. The production of the pro-inflammatory cytokines IL-1β, TNF-α, IFN-α/β and IFN-γ are a driver for inflammation and promotes the development of T helper (Th) 1-type immunity. An intense activation of the Th1-type immunity is associated with tissue damage [30].

The thyme/ivy combination Bronchipret^®^ suppresses bronchoalveolar inflammation and goblet cell hyperplasia in experimental bronchoalveolitis [31]. The tested thyme medications *Thymus vulgaris* mother tincture and Tussamag^®^ inhibited the inflammatory response in the same manner as the thyme/ivy combination Bronchipret^®^, suggesting that the observed anti-inflammatory signal is due to thyme. In A549 cells, the mother tincture, Tussamag^®^ and Bronchipret^®^ induced a significant change in IL-6, IP-10, IFN-λ1, IL-8, IFN-λ2/3 and IFN-β. For Nuli-1 cells, the treatment with these three herbal medications reduced TNF-α, IFN-λ1, IL-12p70, IFN-λ2/3, and IFN-β. Thymol is one of the main bioactive substances in thyme extracts and has been shown to have anti-microbial, anti-oxidant and anti-inflammatory effects in vivo and in vitro [32, 33]. A study done with LPS-activated THP-1 cells demonstrated that thymol could reduce IL-6, IL-8 and IFN-1β mRNA expression [34]. The anti-inflammatory capacity of thymol is attributed to a downregulation of the nuclear factor kappa-light-chain-enhancer of activated B cells (NF-κB) pathway [35].

Our study demonstrated that the ivy medication Prospan^®^ modulate the release of IL-6, TNF-α, GM-CSF, IFN-β in A 549 cells and IFN-λ1, IFN-λ2/3, IFN-β in Nuli-1 cells. These findings align with previous reports, showing that Prospan^®^ reduces the release of IL-6 by inhibiting NF-κB signaling in immune cells, highlighting its anti-inflammatory potential [36, 37]. Interestingly, GM-CSF was significantly increased in NuLi-1 cells after the treatment with Prospan^®^. GM-CSF is mainly known as a pro-inflammatory cytokine, but recent studies also showed an anti-inflammatory and immunosuppressive function, reflecting its complex role [38, 39]. High performance liquid chromatography analysis done by Schulte-Michels revealed that Prospan^®^ cough syrup made from dry ivy leave extract contains the bioactive components rutin, hederacoside C, hederacoside D and α-hederin. Rutin is known to block NF-κB signaling, whereas hederacoside C inhibits TLR2/TLR4 mediated activation of the mitogen-activated protein kinase and NF-κB pathways thereby downregulating IL-6, IL-1β and TNF-α [37, 40].

*Pelargonium sidoides* (*P. sidoides*) is traditionally used in South Africa to treat fever or respiratory diseases [41]. Umckaloabo^®^, an ethanolic root extract of *P. sidoides*, showed an anti-viral effect against influenza A viruses in vivo [42]. Besides its anti-viral properties, *P. sidoides* showed anti-inflammatory activities by downregulating pro-inflammatory cytokines such as IFN-1β, IL-8, TNF-α and C-X-C motif chemokine ligand 10 (CXCL-10 or also IP-10) [43, 44]. Our studies further elucidated the immunomodulatory effect of *P. sidoides*. In A549 and NuLi-1 cells, the TLR7/TLR8-mediated cytokine response was modified. We found that Umckaloabo^®^ treatment reduced the pro-inflammatory cytokines TNF-α, IL-6, IL-8, IL-12p70, IP-10, IFN-γ, IFN-β, IFN-λ1, IFN-λ2/3 and GM-CSF. For IL-6, it was shown that a treatment of Calu-3 cells with Umckaloabo^®^ elevated IL-6 during SARS-CoV-2 infection in vitro [44]. A possible explanation of the divergent IL-6 response observed in our study is the use of different cell lines and experimental settings. Besides IL-6, also IP-10 and IFN-λ3 were identified as predicting the onset of severe symptoms during ongoing COVID-19 infections in patients [45]. The observed downregulation of these cytokines in our study is beneficial and helps to prevent an excessive immune activation and disease progression. A phytochemical fractionation of the *P. sidoides* root extract (Umckaloabo^®^) revealed oligomeric and polymeric prodelphinidins as the main anti-viral constituents, whereas low-molecular fractions containing benzopyranones such as umckalin primarily mediated immunomodulatory and anti-inflammatory effects. The absence of significant effects of cineole observed in our virus mimicry system may be attributed to the used solvent or too low concentrations in the medication we used. In other studies pure cineole has demonstrated anti-inflammatory properties by inhibiting CCL5, IL-6, TNF-α and IL-1β [44].

## Conclusion

In our work, we tested different herbal medications that are commercially available for the treatment of respiratory diseases. By mimicking a viral infection in two different human lung epithelial cells, we could show that almost all tested herbal medications decreased the inflammatory signal that is induced by TLR7 and TLR8. Therefore, they might balance the inflammatory signal during ongoing respiratory viral infections.

## Data Availability

The datasets generated and analysed during the current study are available from the corresponding author on reasonable request.

## Abbreviations

BRO: Bronchipret® liquid TE
°C: Degrees celsius
CXCL-10: C-X-C motif chemokine ligand 10
DMEM: Dulbecco’s modified essential medium
DMSO: Dimethylsulfoxide
EtOH: Ethanol
g: Gravity
Gly.: Glycerol
GM-CSF: Granulocyte-macrophage colony-stimulating factor
h: Hour
IFN: Interferon
IL: Interleukin
IP-10: Interferon-gamma induced protein 10 kD
Lipo: Lipofectamine
Loxo: Loxoribine
ml: Milliliter
NaCl: Sodium chloride
NF-κB: Nuclear factor kappa-light-chain-enhancer of activated B cells
nm: Nanometer
PBS: Phosphate buffered saline
PI: Propidium iodide
PMA: Phorbol-12-myristate-13-acetate
PolyU: Polyuridylic acid
PRO: Prospan® cough drops
*P. sidoides*: *Pelargonium sidoides*
RT: Room temperature
SARS-CoV-2: Severe acute respiratory syndrome coronavirus 2
SD: Standard deviation
SOL: Soledum® capsule forte
ssRNA: Single-stranded ribonucleic acid
Stim.: Stimulated
Th: T helper
TLR: Toll-like receptors
TM: *Thymus vulgaris* mother tincture
TNF: Tumor necrosis factor
TS: Tussamag® concent cough syrup
Triton: Triton X-100
UMC: Umckaloabo® drops
Unstim.: Unstimulated
WST-1: Water-soluble tetrazolium 1
